# Structural Characteristics of Crude Polysaccharides from 12 Selected Chinese Teas, and Their Antioxidant and Anti-Diabetic Activities

**DOI:** 10.3390/antiox10101562

**Published:** 2021-09-30

**Authors:** Huan Guo, Meng-Xi Fu, Ding-Tao Wu, Yun-Xuan Zhao, Hang Li, Hua-Bin Li, Ren-You Gan

**Affiliations:** 1 Research Center for Plants and Human Health, Institute of Urban Agriculture, Chinese Academy of Agricultural Sciences, National Agricultural Science & Technology Center, Chengdu 610213, China; guohuan@caas.cn (H.G.); mxfu_1996@163.com (M.-X.F.); tiantsai@sina.com (H.L.); 2 Institute of Food Processing and Safety, College of Food Science, Sichuan Agricultural University, Ya’an 625014, China; zhaoyunxuan0320@163.com; 3 Key Laboratory of Coarse Cereal Processing, Ministry of Agriculture and Rural Affairs, Sichuan Engineering & Technology Research Center of Coarse Cereal Industralization, School of Food and Biological Engineering, Chengdu University, Chengdu 610106, China; 4 Guangdong Provincial Key Laboratory of Food Nutrition and Health, Department of Nutrition, School of Public Health, Sun Yat-Sen University, Guangzhou 510080, China; lihuabin@mail.sysu.edu.cn

**Keywords:** tea polysaccharides, structural properties, biological properties

## Abstract

Twelve representative edible Chinese teas (*Camellia sinensis* L.) from six categories (dark tea, black tea, oolong tea, white tea, yellow tea, and green tea) were selected in this study. Tea polysaccharides (TPs) were extracted with hot water, and their structural properties and biological activities, mainly antioxidant and anti-diabetic activities, were systematically evaluated. Results revealed that the extraction yields of TPs ranged from 1.81% to 6.38%, and Pu-erh tea polysaccharides had the highest extraction yield (6.38 ± 0.28%). The chemical compositions, molecular weight, and compositional monosaccharides of TPs varied among the six categories of tea. It appeared that all TPs were protein-bound acid heteropolysaccharides, and all TPs exhibited obvious antioxidant and anti-diabetic (e.g., α-glucosidase inhibitory and antiglycation) activities. Particularly, Pu-erh tea polysaccharides also contained the highest total phenolic and protein contents, and also exhibited the best antioxidant and anti-diabetic activities. Moreover, for the structural-function relationship, the heat map analysis found that total phenolic and protein contents in TPs were positively correlated with their antioxidant and anti-diabetic activities, indicating that the presence of phenolic compounds and proteins in the TPs might be the main contributors to their bioactivities. The conclusion from this study can help understand the structural-function relationship of crude tea polysaccharides.

## 1. Introduction

Tea, the product made from the leaves of *Camellia sinensis* L., has become a very popular beverage around the world [1]. Tea has a long history of more than 5000 years as a daily drink. According to the fermentation degrees, tea can be classified into six categories, including dark tea (post-fermented), black tea (deep-fermented), oolong tea (semi-fermented), white tea (mild-fermented), yellow tea (slight-fermented), and green tea (non-fermented) [2]. Previous studies reported that different degrees of fermentation could affect the biological properties of tea [3]. Nowadays, various categories of Chinese tea have been exported to many other countries, such as South Africa, Europe, and North America [4].

Tea has many beneficial functions. The Shen Nong’s Herbal Classic, widely regarded as an ancient and classic book on oriental herbal medicine in China, records the detoxification effect of tea [3]. Recent studies report that drinking tea imparts various nutritional and health functions, such as antioxidation, immuno-regulation, anti-inflammation, anticancer, anti-obesity, anti-diabetes, cardiovascular protection, and hepatoprotection [5,6,7,8]. These health functions can be mainly attributed to various bioactive components in tea, such as polyphenols (e.g., catechins and flavonoids), polysaccharides, pigments, alkaloids (theobromine, caffeine, theophylline, etc.), free amino acids, saponins, and inorganic elements [9]. Polyphenols, especially catechins, are the focus of most research about tea. However, polysaccharides, as another type of main bioactive ingredients in tea, have been overlooked. Water-soluble tea polysaccharides are widely regarded as powerful natural antioxidants and have beneficial effects on glucose homeostasis and improve insulin resistance in type 2 diabetes [10]. In recent reports, tea polysaccharides have been identified as acidic polysaccharides with mineral elements and proteins [11], and the molecular weight and compositional monosaccharides of tea polysaccharides varied with different tea categories, drying methods, and processing methods [12]. In addition, tea polysaccharides have also been proved to have many biological properties, such as anti-radiation, immunological, anti-cancer, and anticoagulant activities [3,13,14,15]. Wang et al. reported that crude tea polysaccharides combined with tea polyphenols showed stronger antioxidant activities than purified tea polysaccharides [16].

Therefore, in this study, the structural properties and biological activities, mainly antioxidant and anti-diabetic activities, of crude polysaccharides from 12 selected representative Chinese teas from six categories (dark tea, black tea, oolong tea, white tea, yellow tea, and green tea) were systematically evaluated and compared. The results can be helpful for further application of tea polysaccharides in the development of functional food in the food industry.

## 2. Materials and Methods

### 2.1. Materials and Chemicals

Twelve representative Chinese teas (*Camellia sinensis* L.) from six categories were purchased from the local market in Chengdu, China. The basic information of the 12 kinds of tea is presented in Table 1. The tea samples were dried, crushed, passed through a 60-mesh screen, and stored at −20 °C.

Chemicals, including arabinose, glucose, galactose, rhamnose, mannose, xylose, glucuronic acid, galacturonic acid, butylated hydroxytoluene (BHT), vitamin C, sodium azide, 1-phenyl-3-methyl-5-pyrazolone (PMP), 2,2-diphenyl-1-picrylhydrazyl (DPPH), 3-ethylbenzthiazoline-6-sulphonic acid (ABTS), 4-nitrophenyl *β*-D-glucopyranoside (pNPG), acarbose, and α-glucosidase reagent (10 U/mg) were purchased from Sigma-Aldrich (MO, USA). Thermostable α-amylase reagent (40000 U/g) was purchased from Solarbio (Beijing, China), and all reagents and chemicals were of analytical grade.

### 2.2. Extraction of Crude Polysaccharides from 12 Chinese Teas

Twelve crude tea polysaccharides were extracted based on our reported methods [17]. To be brief, tea powder (2.0 g) was precisely weighed, and followed by an ultrasonic cleaner (PL-S80T, Kangshijie Biotechnology Co., Ltd., Dongguan, China; 800 W) to treat the powder with 80% ethanol and the power of 640 W twice for 30 min to eliminate most small molecules. Then, samples were continuously shaken for 2 h at 95 °C with distilled water (1:20, *w/v*) to extract crude tea polysaccharides (TPs), which was repeated twice. Besides, the extraction yields were determined by the weighing method. The total polysaccharides, protein content, total phenolic content (TPC), and uronic acids in TPs were measured by the phenol-sulfuric acid method, Bradford’s method, the Folin-Ciocalteu method, and m-hydroxydiphenyl method, respectively, based on our previous studies [17,18].

### 2.3. Test of Structural Properties of TPs

#### 2.3.1. Test of Molecular Weights (*M_w_*), Polydispersities (*M_w_/M_n_*), and Compositional Monosaccharides

High-performance size-exclusion chromatography added with multi-angle laser light scattering and refractive index detector (HPSEC-RID, Wyatt Technology Co., Santa Barbara, CA, USA) was employed to estimate the weight-average *M_w_* and *M_w_/M_n_* of TPs. The application of the Shodex OHpak SB-806M HQ column was made at 30 °C. The concentration of tea polysaccharide was 1.0 mg/mL, and the injection volume was 100 μL. Besides, high-performance liquid chromatography (HPLC, Agilent Technologies, Santa Clara, CA, USA) exploration was used to measure constituent monosaccharides of TPs on the basis of the approach reported in the past [19].

#### 2.3.2. Fourier Transform Infrared (FT-IR) Spectroscopy and Nuclear Magnetic Resonance (NMR) Analysis

A Nicolet iS10 FT-IR (ThermoFisher Scientific, Waltham, MA, USA) was employed to conduct the FT-IR spectroscopy analysis of TPs at a frequency range of 400–4000 cm^−1^. The degree of esterification (DE) was measured by the band regions at 1600–1650 cm^−1^ (free uronic acids) and 1700–1750 cm^−1^ (esterified uronic acids). The estimation of DE was made on the basis of the equation below [18],

$$\mathrm{DE}\left(\%\right)=\left(\frac{A_{1735}}{A_{1735}{+A}_{1630}}\right)\;\times \;100\%$$

The NMR exploration of TPs was conducted on the basis of the approach reported in the past by Nie et al. [20]. To be brief, the storage of 0.5 mL of deuterated water (D_2_O) with 20.0 mg of the sample was made at room temperature (25 ± 1 °C) overnight before the NMR analysis. Besides, a Bruker Ascend 600 MHz spectrometer (Bruker, Rheinstetten, Germany) configured with a z-gradient probe with frequencies of 600.13 MHz for proton and 150.90 MHz for carbon was used to measure the 1D NMR spectra (^1^H and ^13^C).

### 2.4. In Vitro Antioxidant Assays

Our previously reported assays [21] were employed to measure the reducing power, DPPH, and ABTS radical scavenging activity of TPs.

Determination of the reducing power of TPs. TPs (100 µL) at five concentrations were mixed with 100 µL of 1% potassium ferricyanide (*w/v*) in 20 mM PBS (pH 6.8), and the mixture was incubated for 20 min at 50 °C. Then, 100 µL of 10% trichloroacetic acid (*w/v*) was added to the mixture to incubate for another 10 min. Finally, 20 µL of 0.1% ferric chloride (*w/v*) was added, and the absorbance was determined at 700 nm after 30 min.

Determination of the DPPH radical scavenging activity of TPs. Each sample (20 µL) at five concentrations was added to 200 µL of 0.35 mM ethanolic DPPH solutions. Then, the mixture was incubated at 37 °C for 30 min in the dark with shaking, and the absorbance was determined at 517 nm.

Determination of the ABTS radical scavenging activity of TPs. The ABTS radical cation solution was produced by the interaction of 2.45 mM potassium persulphate and 7 mM ABTS at room temperature for 16 h in the dark. The ABTS radical solution was diluted with phosphate buffer (0.2 M, pH 7.4) to obtain an absorbance of 0.750 ± 0.02 at 734 nm. Each sample (20 µL) at five concentrations was added to 200 µL of ABTS radical solution, and the mixture was incubated for 20 min at 30 °C. The absorbance was measured at 734 nm.

In addition, BHT as the positive control for the DPPH assay, and Vc as the positive control for the decreasing power and ABTS assays. Finally, the calculation of IC_50_ values (µg/mL) was made through a log-regression curve.

### 2.5. Evaluation of the Anti-Diabetic Activity of TPs

#### 2.5.1. In Vitro α-Glucosidase Inhibitory Assay

The analysis of *α*-glucosidase inhibitory assays of TPs was made at five concentrations according to the approach reported in the past [20]. Acarbose was applied as the positive control. Then, the calculation of IC_50_ values (µg/mL) was made through a log-regression curve.

#### 2.5.2. In Vitro Antiglycation Assay

The bovine serum albumin-glucose model (BSA-Glc) was employed to determine the inhibition on the generation of advanced glycation end-products (AGEs) by TPs as the approach reported in the past [22]. The measurement of AGEs analysis of each sample was made at five different concentrations ranging from 0.25 to 4.00 mg/mL. Fluorescence at the wavelength of 370 nm was adopted to quantify AGEs for excitation and 440 nm for emission. The application of AG as the positive control was made. Then, the calculation of IC_50_ values (mg/mL) was made through a log-regression curve.

### 2.6. Statistical Analysis

Origin 9.0 software (OriginLab, Northampton, MA, USA) was applied for the statistical analysis, which was conducted by one-way analysis of variance (ANOVA) plus *post hoc* Duncan’s test, with *p* < 0.05 defined as statistical significance. Origin 9.0 software was also applied for the heat map drawing, and the correlation coefficient (Pearson *r*) was calculated. All the assays were carried out in triplicate, and data were presented as means ± standard deviation.

## 3. Results

### 3.1. The Extraction Yields and Chemical Compositions of TPs

Table 2 summarizes the yields and chemical compositions of 12 TPs. The extraction yields of TPs ranged from 1.81% to 6.38%. TP-4 had the highest extraction yield (6.38 ± 0.28%), followed by TP-3, TP-5, and TP-6, which had similar extraction yields. In addition, TP-1 had the lowest extraction yields than other TPs, which was estimated to be 1.81%. Furthermore, the contents of total polysaccharides in TPs ranged from 63.51% to 88.44%, suggesting that polysaccharides were the main composition in each sample.

The contents of proteins in TPs ranged from 3.42% to 11.73%, and TP-4 had a significantly (*p* < 0.05) higher protein content (11.73 ± 0.76%) but significantly (*p* < 0.05) lower total polysaccharide content (63.51 ± 0.76%) compared to other TPs. These results suggested that all the tested TPs were polysaccharide-protein complexes, which was consistent with previous studies [23]. On the other hand, despite 80% ethanol was used to remove most of the small molecules, it was found that some phenolic compounds could still be detected as indicated by the TPC assay. TPC in TPs ranged from 11.64 to 162.43 mg GAE/g. Compared with other TPs, significantly (*p* < 0.05) higher TPC was found in TP-4 (162.43 ± 9.43 mg GAE/g) and TP-1 (92.88 ± 7.34 mg GAE/g). The total uronic acid content of TPs ranged from 16.04% to 47.39%. TP-5 (47.39 ± 1.12%) and TP-6 (44.27 ± 1.07%) had the highest uronic acid content, while TP-1 (16.04 ± 0.70%) and TP-2 (18.33 ± 0.65%) had the lowest uronic acid content. Overall, the results indicated that all TPs might be acid heteropolysaccharides, probably bound with polyphenols. Previous studies reported that tea polysaccharides were mostly glycoconjugates, which not only contain monosaccharides but also connect with protein, uronic acid, polyphenols, inorganic elements, etc [3].

### 3.2. Structural Properties of TPs

#### 3.2.1. Molecular Weights of TPs

**Figure 1:**
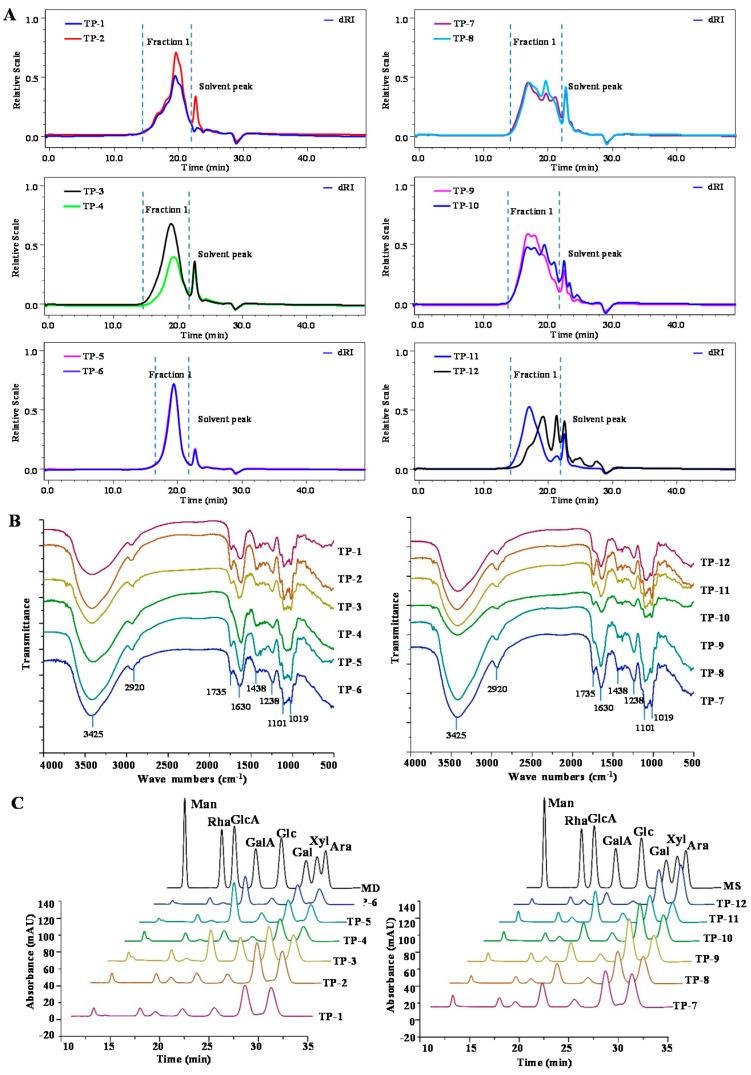
HPSEC chromatograms (**A**), FT-IR spectra (**B**), and HPLC profiles (**C**) of TPs. TPs 1–12 represent tea polysaccharides as shown in Table 1. Abbreviations: MS, mixed standards of monosaccharides; Man, Rha, GlcA, GalA, Glc, Gal, Xyl, and Ara indicate mannose, rhamnose, glucuronic acid, galacturonic acid, glucose, galactose, xylose, and arabinose, respectively.

Figure 1A displays the HPSEC-RID chromatograms of TPs, and the weight-average *M_w_* and *M_w_/M_n_* of polysaccharides in TPs are presented in Table 2. The *M_w_* of TPs varied from 9.16 × 10^4^ to 73.34 × 10^4^ Da, and the polydispersities of TPs ranged from 1.47 to 3.53, consistent with their HPSEC chromatograms.

The 12 kinds of tea showed remarkable differences in molecular weights of polysaccharides. TP-9 (73.34 × 10^4^ Da) had the highest *M_w_*, while TP-5 (9.16 × 10^4^ Da) and TP-6 (12.48 × 10^4^ Da) had the lowest *M_w_*. The difference in *M_w_* distribution may be due to the different categories and production places of the 12 kinds of tea. Besides, previous studies reported that different fermentation degrees had a significant effect on the *M_w_* of tea polysaccharides. According to Chen et al. [24], the *M_w_* of polysaccharides was reduced with the increase of the fermentation degree (e.g., black tea > oolong tea > green tea). The reason for this phenomenon was due to the hydrolysis of the carbohydrate by the existing enzymes in tea. However, the current study found that two semi-fermented oolong tea polysaccharides, TP-5 and TP-6, showed the lowest molecular weight, inconsistent with the above study, and the reason may be caused by different fermentation degrees, categories, and production place of different kinds of tea.

#### 3.2.2. FT-IR Spectra of TPs

FT-IR spectra were applied to compare the preliminary structural properties of TPs. As shown in Figure 1B, the FT-IR spectra of TPs exhibited typical absorption peaks of polysaccharides in the wavelength range from 4000 to 400 cm^−1^. Broad peaks at 3425 cm^−1^ and 2920 cm^−1^ were caused by the O–H bond stretch and the characteristic absorption of the C-H bond, respectively. Meanwhile, the absorption peaks at approximately 1438 and 1238 cm^−1^ were attributed to C−H/O−H and −OCH_3_, respectively. The absorption peaks at approximately 1101 and 1019 cm^−1^ were assigned to the absorption bands of pyran-glycosides. These results suggested that all TPs were composed of polysaccharides and proteins, in agreement with the chemical composition results and previous studies [25,26]. Although the main absorption peaks of TPs were consistent, there were differences in some absorption peaks. Except for the TP-4, the absorption peak at approximately 1735 cm^−1^ was detected to be attributed to the tensile vibration of the esterified carboxyl group [27]. Besides, as shown in Table 2, except for the degree of esterification (DE) of TP-4 was not detected, other TPs had the DE ranging from 8.99% to 46.46%, which was in agreement with the FT-IR spectra of TPs. As reported by Mao et al. [28], the FT-IR spectra of Pu-erh tea polysaccharides showed that the absorption peak of the esterified carboxyl group was not detected, in agreement with the results of this study. The reason for this phenomenon might be the differences in tea varieties and tea-making processes.

#### 3.2.3. Compositional Monosaccharides of TPs

The compositional monosaccharides of TPs were further investigated. Figure 1C shows the HPLC-UV profiles of TPs, and the molar ratios of monosaccharides in TPs are summarized in Table 3. The main compositional monosaccharides of TPs were Man, Rha, GlcA, GalA, Glc, Gal, and Ara. In addition, for TP-1, TP-2, TP-5, TP-7, TP-8, TP-9, TP-10, and TP-12, a small amount of Xyl was detected by HPLC-UV. Wang et al. reported that oolong tea polysaccharides were composed of seven monosaccharides, and the highest content of monosaccharides was Glc, Gal, and Ara [26]. Chen et al. [24] reported that there were significant differences in the composition and content of the monosaccharides in black tea, oolong tea, and green tea. Results suggested that the compositional monosaccharides and the molar ratios of monosaccharides of TPs were significantly different with different categories, production place, and fermentation degrees.

#### 3.2.4. NMR Analysis

**Figure 2:**
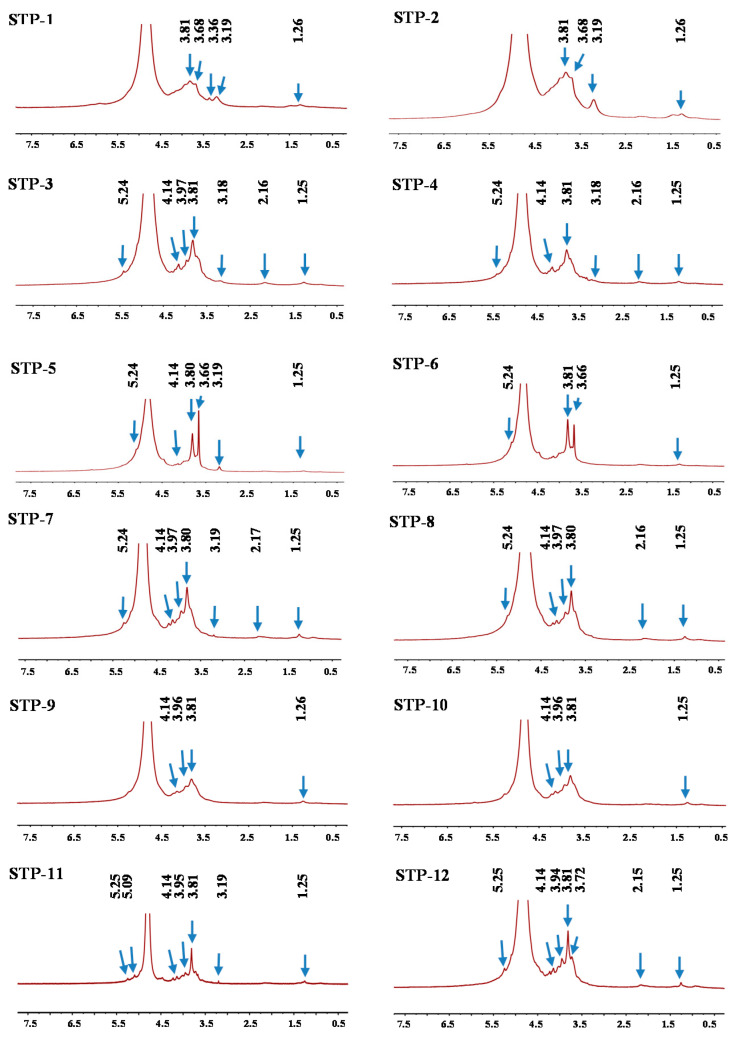
^1^H NMR spectra of TPs. TPs 1–12 represent tea polysaccharides as shown in Table 1.

**Figure 3:**
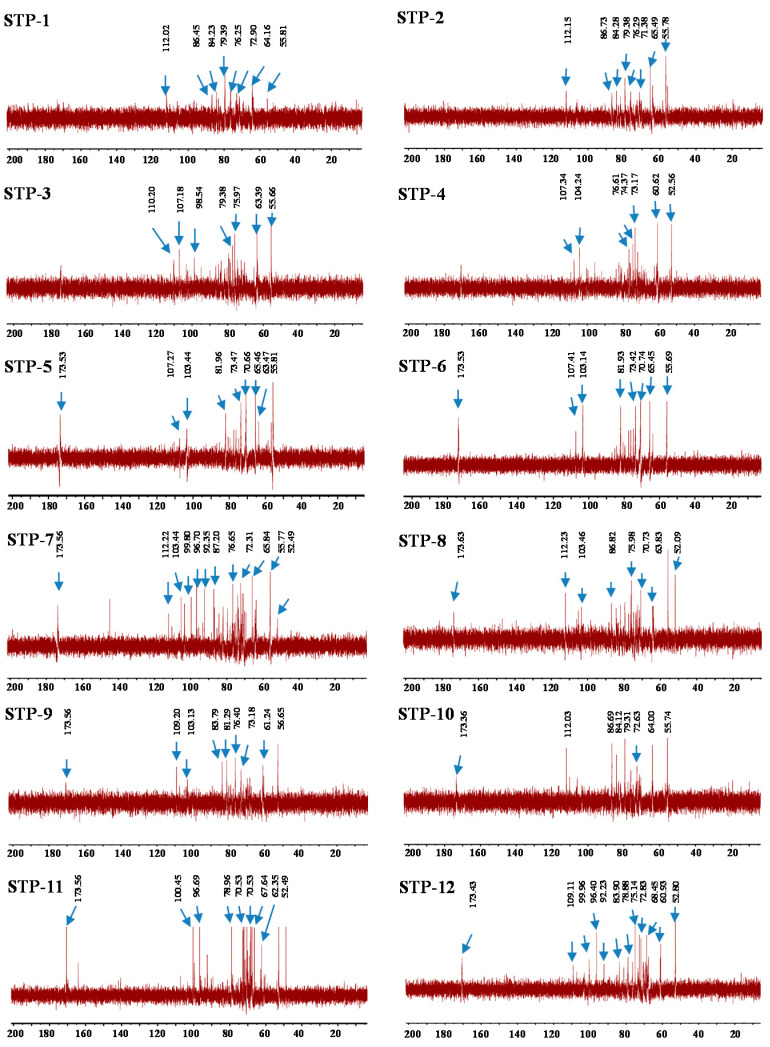
^13^C NMR spectra of TPs. TPs 1–12 represent tea polysaccharides as shown in Table 1.

The structural properties of TPs were compared by applying NMR spectra. ^1^H and ^13^C explorations (1D NMR spectra) are displayed in Figure 2 and Figure 3. Apparently, the NMR spectra of tea polysaccharides with the same degree of fermentation were similar. In general, the signals at 5.25 and 1.25 ppm were tentatively attributed to the H-1 and H-6 of 1,2-*α*-L-Rha [27], respectively. The peaks at 5.09 and 4.14 ppm were tentatively attributed to the H-1 and H-2 of 1,5-*α*-L-Ara [27], respectively. The weak signal at 2.16 ppm was tentatively attributed to the presence of acetyl groups [27], and the strong peak at 3.80 ppm was tentatively attributed to the signal of methyl esters which connect to carboxyl groups of D-GalA [27]. The peak at 3.96 ppm was tentatively attributed to the H-3 of 1,4-*α*-D-Glc [27]. The signals at 3.36 and 3.19 ppm were temporarily ascribed to be the H-3 and H-2 of 1,4-*β*-D-Xyl [29]. Comparing to other TPs, the signal at 3.66 ppm was ascribed in TP-5 and TP-6, which was tentatively attributed to the H-2 of 1,4-*α*-D-GalA [30]. However, some anomeric peaks were not presented in the ^1^H-NMR spectra. The reason for this result might be that the ^1^H NMR spectra (Figure 2) showed a very wide peak of deuterated water (D_2_O) located from 4.6 ppm to 5.0 ppm, therefore, several peaks related to the anomeric area might be covered by D_2_O.

As shown in Figure 3, the ^13^C NMR spectra of TPs showed that the C-6 of un-esterified carbonyl groups of D-GalA was at 173.56 ppm [27]. However, comparing to other TPs, the signal at approximately 173.56 ppm was not ascribed in TP-4 and TP-1. Besides, the signal detected at approximately 103.44 ppm in some samples was tentatively attributed to the C-1 of 1,2-*α*-L-Rha [31]. The signal at approximately 64.16 ppm was tentatively attributed to the C-6 of 1, 4-*β*-D-Man [27]. The signals at approximately 65.46 and 99.80 ppm were tentatively attributed to the C-6 of 1,2-*α*-D-Ara and C-1 of 1,2-*α*-L-Rha [32,33]. The signal at approximately 76.65 ppm was tentatively attributed to the C-3 of 1,4-*α*-D-Glc [34]. The C-4 signal for 1, 4-*α*-D-Gal was at approximately 79.39 ppm [35]. The C-1 signals for 1, 3-*α*-L-Ara and 1, 5-*α*-L-Ara were at approximately 112.02 and 110.20 ppm, respectively, and the signal at approximately 86.45 ppm was tentatively attributed to the C-2 of 1, 5-*α*-L-Ara [36]. The signals at approximately 73.47 and 107.18 ppm were tentatively attributed to the C-2 and C-1 of 1, 4-*β*-D-Gal [33,37]. The signal at approximately 52.49 ppm was the response to the existence of a methyl group esterified carboxyl group of GalA [27]. Furthermore, the peaks between 52 and 57 ppm were tentatively attributed to the amino-substituted carbon signals of an amino sugar residue [38], indicating the existence of proteins in the sample.

Generally, outcomes from the compositional monosaccharides, the FT-IR spectra, and the NMR spectra indicated that all TPs were protein-bound acid heteropolysaccharides. Nevertheless, the precise structures of TPs need further clarification (e.g., applying 2D NMR and methylation) in the future. Furthermore, to study the structure-activity relationship between the structural characteristics of polysaccharides and their bioactivities, it is necessary to further purify polysaccharides and analyze their detailed structural characteristics.

### 3.3. In Vitro Antioxidant Activities

**Figure 4:**
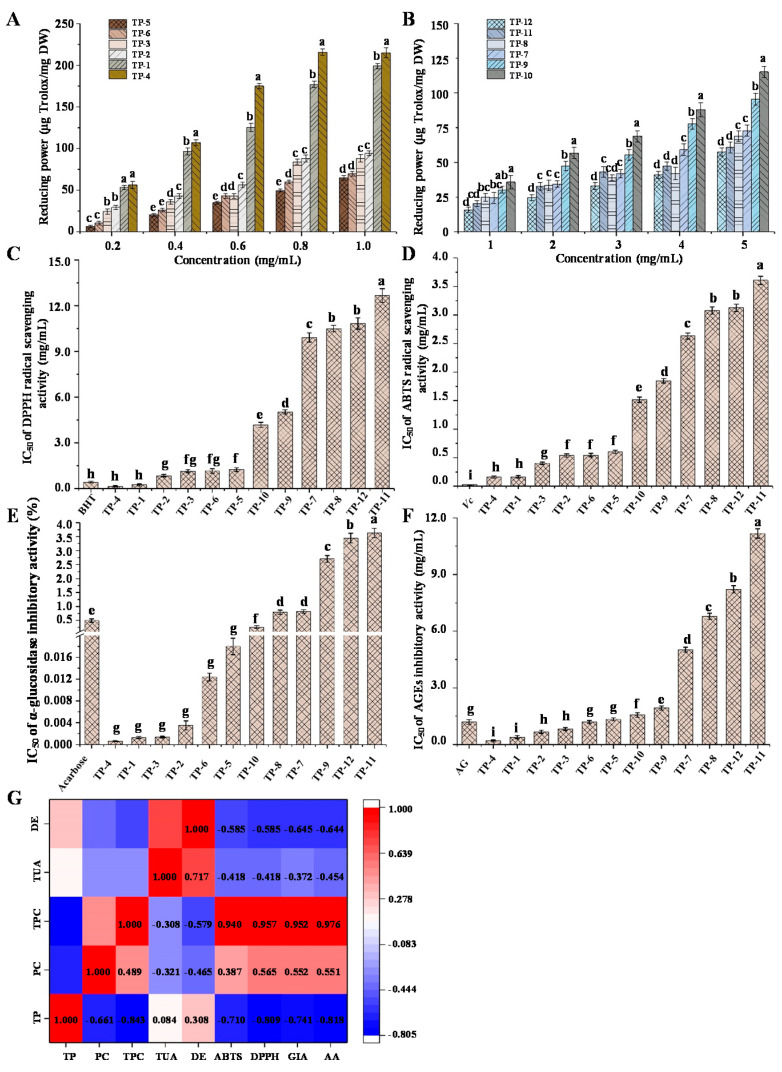
Reducing power (**A**,**B**), DPPH radical scavenging activity (**C**), ABTS radical scavenging activity (**D**), *α*-glucosidase inhibitory activity (**E**), and in vitro antiglycation activity (**F**) of TPs, and heat map analysis of the correlation between chemical composition and biological properties (**G**). TPs 1–12 represent tea polysaccharides as shown in Table 1. The error bars indicated standard deviation, and statistical analysis was carried out by ANOVA plus *post hoc* Ducan’s test, and statistical significance (*p* < 0.05) was indicated with different lowercase letters (a–i). Abbreviations: BHT, butylated hydroxytoluene; Vc, vitamin C; AG, aminoguanidine; TP, PC, TPC, TUA, DE, GIA, and AA indicate total polysaccharides, protein contents, total phenolic content, total uronic acids, degree of esterification, *α*-glucosidase inhibitory activity, and antiglycation activity, respectively.

Free radical-induced oxidative stress is one of the significant reasons resulting in different diseases including cancer, neurodegenerative disorders, and inflammatory diseases [39]. According to previous researches, polysaccharides could block ROS-induced oxidative damage through scavenging free radicals [40]. Hence, the investigation into antioxidant activities of TPs was made. In the present study, the reducing powers, DPPH, and ABTS radical scavenging capacities were used to evaluate the in vitro antioxidant activities of TPs. The reducing powers of TPs are shown in Figure 4A,B. The DPPH and ABTS radical scavenging activities of TPs are shown in Figure 4C,D, respectively. In this assay, the concentration-dependent profile of reducing powers as well as DPPH and ABTS radical scavenging activities was obvious for all TPs. At the concentration of 1.0 mg/mL, TP-4 (214.91 μg Trolox/mg) and TP-1 (198.81 μg Trolox/mg) had the highest reducing powers, while TP-11 (20.27 μg Trolox/mg) and TP-12 (15.74 μg Trolox/mg) had the lowest reducing powers. Besides, the IC_50_ values of DPPH and ABTS radical scavenging activities of TPs ranged from 0.14 to 12.66 mg/mL and 0.16 to 3.61 mg/mL, respectively. The IC_50_ values of DPPH and ABTS radical scavenging activities of the positive controls were 0.42 mg/mL (BHT) and 0.03 mg/mL (Vc), respectively. Compared with the previous reports about polysaccharides, the current study found that most TPs exhibit higher antioxidant activities [18,21]. These results indicated that tea polysaccharides had the potential to relieve oxidative stress by scavenging free radicals. Moreover, the dark tea (TP-4 and TP-3) and the black tea (TP-1 and TP-2) possessed the highest antioxidant activities among all tested TPs, while TP-11 and TP-12, two yellow tea polysaccharides, showed the lowest antioxidant activities. Notably, Pu-erh tea polysaccharides (TP-4) had the highest level of total phenolics and antioxidant activities.

The correlation between chemical composition and biological properties of TPs was evaluated by heat map analysis. As shown in Figure 4G, there were positive correlations of the TPC with DPPH (Pearson *r* = 0.957) and ABTS (Pearson *r* = 0.940) radical scavenging activities, suggesting that the presence of phenolic compounds in the TPs might be the main contributor to their antioxidant activities. In addition, the protein content had moderate correlations with DPPH (Pearson *r* = 0.565) and ABTS (Pearson *r* = 0.387) radical scavenging activities, indicating that proteins in the TPs might also contribute to their antioxidant activities. This result is consistent with a previous study that protein was positively correlated with antioxidant activities of mushrooms polysaccharides [41]. Moreover, the current study found that polyphenols might be the major antioxidant component in the TPs, which was consistent with the result of Alasalvar et al. [42]. Fan et al. also reported that the antioxidant activities of crude tea polysaccharides are connected with the tea polyphenol content, protein content, and the synergistic effect of tea polysaccharides and polyphenols [43]. Indeed, many small molecule compounds, especially the hydrolysable tannins and flavonoids from tea, were also considered to be the major components with excellent antioxidant activities [44]. In short, different kinds of tea had great effects on the antioxidant activities of TPs, especially, the dark tea and black tea polysaccharides exhibit excellent antioxidant activities, which might be associated with the complex alterations in the chemical compositions of tea that occurred during different fermentation degrees, leading to changes in their biological characteristics.

### 3.4. In Vitro Anti-Diabetic Activities of TPs

#### 3.4.1. In Vitro α-Glucosidase Inhibitory Activity

Diabetes mellitus (DM), one of the key public health challenges in the 21st century, has a serious impact on human health. Over the past several decades, oral hypoglycemic agents (OHA) have been widely used to treat Type 2 DM. However, recent studies suggest that OHA may cause many adverse effects, including weight gain, hypoglycemia, lactic acidosis, gastrointestinal disturbance, and fluid retention [24,45]. Therefore, it is necessary for the exploration of safe, green, and high-efficient OHA alternatives. Tea has been widely used as a health drink to prevent and treat hyperglycemia. It is speculated that TPs may have a hypoglycemic effect. Therefore, the *α*-glucosidase inhibitory activity of TPs was further investigated. As shown in Figure 4E, the IC_50_ values of *α*-glucosidase inhibition by TPs ranged from 4 × 10^−4^ to 3.63 mg/mL. For the positive control, the IC_50_ value of the *α*-glucosidase inhibitory activity of acarbose was determined to be 0.50 mg/mL, and it was further confirmed that TP-4, TP-1, TP-3, TP-2, TP-6, TP-5, and TP-10 exhibited stronger *α*-glucosidase inhibitory activities than the positive control.

As shown in Figure 4G, the *α*-glucosidase inhibitory activity was positively correlated with TPC (Pearson *r* = 0.952) and protein content (Pearson *r* = 0.552), suggesting that the presence of phenolic compounds and protein in the TPs might also mainly contribute to their *α*-glucosidase inhibitory activity. Mao et al. reported that the protein content of tea polysaccharides was positively associated with their antioxidant activity and *α*-glycosidase inhibitory effect [28]. Meanwhile, the higher protein content enhanced the interaction with other molecules, which may be an important factor affecting the biological properties of tea polysaccharides [28]. Notably, the superior *α*-glycosidase inhibitory activity of dark tea polysaccharides (TP-4) and black tea polysaccharides (TP-1) were not only related to their categories and production place, but also related to the fermentation time. Xu et al. [23] reported that the *α*-glycosidase inhibitory capacity and antioxidant capacity of fermented tea might increase with the fermentation time. The prolonged fermentation might result in the polymerization of polysaccharides and proteins, changing the conformation and configuration of the proteins and further improving the bioactivities of polysaccharides [23].

#### 3.4.2. In Vitro Antiglycation Activity

Previous studies showed that the free amino groups in proteins and fats could combine with the reducing sugar, leading to the formation of AGEs [46]. AGEs cause cell damage at various levels and contribute substantially to the progression of diabetes and its complications, such as cataracts, cancer, aging, neurodegenerative diseases, and cardiovascular diseases [47]. Reducing the generation of AGEs may be a feasible method to prevent or alleviate the onset of diabetes and its complications. Therefore, the antiglycation activity of TPs was further investigated and compared. As shown in Figure 4F, the IC_50_ values of antiglycation activity of TPs ranged from 0.20 to 11.17 mg/mL. Apparently, compared with the positive inhibitor AG (IC_50_ = 1.20 mg/mL), TP-4, TP-1, TP-2, and TP-3 also showed excellent antiglycation activities. Furthermore, TP-4 had the highest inhibitory activity on the formation of AGEs with the IC_50_ value of 0.20 ± 0.05 mg/mL. Besides, TP-11 and TP-12 had the lowest inhibitory activity. As mentioned above, different categories, production places, and fermentation times could affect the chemical composition and structural characteristics of TPs, including protein content, TPC, and molecular weight, etc. According to Figure 4G, the antiglycation activity was positively correlated with TPC (Pearson *r* = 0.976) and protein content (Pearson *r* = 0.551), suggesting that the presence of phenolic compounds and proteins in the TPs might also mainly contribute to their antiglycation activities. In addition, TP-4 with medium molecular weight showed the optimal bioactivities, demonstrated that the molecular weight might also influence the bioactivities of TPs. Xu et al. [23] reported that the polysaccharides of black tea showed the best biological properties among the three kinds of tea (green tea, oolong tea, and black tea). It was demonstrated that the fermentation degree of tea could improve its bioactivities. As reported by Lv et al. [44], a series of chemical reactions, such as oxidation, degradation, and condensation, occurred in the fermentation process of tea. During this process, new substances were formed and provided unique flavor and beneficial effects. Therefore, the activities of polysaccharides may be influenced by a combination of different factors, including protein content, TPC, and molecular weight, which was consistent with previous reports [48,49,50,51]. As a summary, these results provided a strong basis for explaining the hypoglycemic mechanism of TPs and the application of TPs in functional foods and medicine.

## 4. Conclusions

In this study, the structural characteristics, in vitro antioxidant activities, and in vitro anti-diabetic activities of 12 selected TPs were systematically evaluated. Results revealed that the main compositional monosaccharides of TPs were Man, Rha, GlcA, GalA, Glc, Gal, and Ara. The *M_w_* of TPs varied from 9.16 × 10^4^ to 73.34 × 10^4^ Da. All TPs from different kinds of teas were protein-bound acid heteropolysaccharides, and all TPs exhibited obvious biological properties, including antioxidant activities and anti-diabetic activities. Particularly, Pu-erh tea polysaccharides had the highest contents of TPC and protein, as well as the most excellent biological properties. Moreover, it was speculated that the presence of phenolic compounds and proteins in the TPs might be the main contributor to their biological properties. In general, dark tea and black tea polysaccharides exhibited stronger antioxidant, *α*-glucosidase inhibitory, and antiglycation activities than other selected tea polysaccharides. The results of this study could help understand the relationship among the chemical composition, structural properties, and biological properties of natural polysaccharides. The Pu-erh tea polysaccharides, exhibit the best biological activities among selected tea polysaccharides, have the potential to be developed into functional food for the prevention and treatment of certain chronic diseases, such as diabetes.

## Figures and Tables

**Table 1:** Basic information about the 12 selected Chinese teas.

No.	Tea Name	Production Place	Fermentation Degree	Category
TP-1	Dianhong Congou Black Tea	Kunming, Yunnan	Deep-fermented	Black tea
TP-2	Yichang Congou Black Tea	Yichang, Hubei	Deep-fermented	Black tea
TP-3	Fuzhuan Brick Tea	Anhua, Hubei	Post-fermented	Dark tea
TP-4	Pu-erh Tea	Pu’er, Yunnan	Post-fermented	Dark tea
TP-5	Fenghuang Shuixian Tea	Chao’an, Guangdong	Semi-fermented	Oolong tea
TP-6	Wuyi Rock Tea	Wuyishan, Fujian	Semi-fermented	Oolong tea
TP-7	Dianqing Tea	Kunming, Yunnan	Non-fermented	Green tea
TP-8	Lushan Yunwu Tea	Jiujiang, Jiangxi	Non-fermented	Green tea
TP-9	Gongmei White Tea	Nanping, Fujian	Mild-fermented	White tea
TP-10	White Peony Tea	Nanping, Fujian	Mild-fermented	White tea
TP-11	Weishan Maojian Tea	Ningxiang, Hunan	Light-fermented	Yellow tea
TP-12	Yuan’an Luyuan Tea	Yichang, Hubei	Light-fermented	Yellow tea

**Table 2:** Chemical compositions of TPs.

No.	Extraction Yields (%)	Total Polysaccharides (%)	Protein Contents (%)	Degree of Esterification (%)	Total Uronic Acids (%)	TPC (mg GAE/g)
TP-1	1.81 ± 0.12 ^f^	78.97 ± 1.01 ^de^	5.23 ± 0.34 ^de^	12.43 ± 0.45 ^g^	16.04 ± 0.70 ^i^	92.88 ± 7.34 ^b^
TP-2	2.37 ± 0.19 ^e^	83.26 ± 0.93 ^b^	3.42 ± 0.31 ^g^	15.94 ± 0.69 ^f^	18.33 ± 0.65 ^h^	53.41 ± 5.92 ^d^
TP-3	5.65 ± 0.23 ^b^	82.39 ± 0.88 ^bc^	5.05 ± 0.42 ^de^	25.16 ± 0.72 ^e^	42.71 ± 0.99 ^c^	65.50 ± 5.59 ^c^
TP-4	6.38 ± 0.28 ^a^	63.51 ± 0.76 ^g^	11.73 ± 0.76 ^a^	-	20.57 ± 0.42 ^g^	162.43 ± 9.43 ^a^
TP-5	5.49 ± 0.35 ^b^	75.31 ± 1.09 ^f^	5.30 ± 0.45 ^de^	35.37 ± 0.51 ^c^	47.39 ± 1.12 ^a^	51.31 ± 7.86 ^d^
TP-6	5.31 ± 0.29 ^b^	77.13 ± 1.22 ^ef^	4.71 ± 0.43 ^de^	36.68 ± 0.46 ^b^	44.27 ± 1.07 ^b^	55.41 ± 4.55 ^d^
TP-7	3.05 ± 0.21 ^cd^	88.44 ± 1.36 ^a^	3.84 ± 0.30 ^fg^	34.59 ± 0.55 ^c^	33.36 ± 0.74 ^d^	13.54 ± 3.05 ^fg^
TP-8	3.26 ± 0.22 ^c^	86.87 ± 1.73 ^a^	4.46 ± 0.36 ^ef^	8.99 ± 0.45 ^h^	33.15 ± 0.98 ^d^	14.84 ± 2.67 ^fg^
TP-9	3.09 ± 0.22 ^cd^	82.22 ± 1.28 ^bc^	7.96 ± 0.52 ^c^	30.73 ± 0.44 ^d^	29.96 ± 0.86 ^e^	20.83 ± 2.62 ^ef^
TP-10	2.58 ± 0.27 ^e^	81.83 ± 1.65 ^bc^	3.60 ± 0.39 ^g^	31.80 ± 0.69 ^d^	27.68 ± 0.63 ^f^	25.63 ± 2.83 ^e^
TP-11	2.69 ± 0.18 ^de^	80.49 ± 1.66 ^cd^	5.45 ± 0.42 ^d^	46.46 ± 0.80 ^a^	42.27 ± 0.82 ^c^	11.64 ± 1.31 ^g^
TP-12	3.23 ± 0.25 ^c^	82.35 ± 1.48 ^bc^	9.34 ± 0.67 ^b^	15.31 ± 0.37 ^f^	20.37 ± 0.59 ^g^	12.34 ± 1.88 ^g^

TPs 1–12 represent tea polysaccharides as shown in Table 1. Values represent mean ± standard deviation, and statistical analysis was carried out by ANOVA plus *post hoc* Ducan’s test, and statistical significance (*p* < 0.05) was indicated with different lowercase letters (a–i).

**Table 3:** Molecular weight (*M_w_*), polydispersity (*M_w_/M_n_*), and molar ratios of compositional monosaccharides of TPs.

	*M_w_* × 10^4^ (Da)	*M_w_*/*M_n_*	Monosaccharides and Molar Ratios							
			Man	Rha	GlcA	GalA	Glc	Gal	Xyl	Ara
TP-1	21.20 (± 0.66%) ^gh^	2.48 (± 1.07%)	0.17	0.26	0.21	0.82	0.30	1.05	0.04	1.00
TP-2	23.88 (± 1.35%) ^e^	1.47 (± 1.90%)	0.19	0.28	0.24	1.01	0.28	1.23	0.05	1.00
TP-3	27.69 (± 0.44%) ^d^	2.19 (± 0.67%)	0.19	0.39	0.15	3.71	0.90	1.26	-	1.00
TP-4	20.24 (± 0.60%) ^h^	1.93 (± 0.98%)	0.39	0.51	0.12	2.41	0.34	1.49	-	1.00
TP-5	9.16 (± 0.62%) ^k^	2.19 (± 1.00%)	0.18	0.41	0.11	7.41	0.56	1.24	0.06	1.00
TP-6	12.48 (± 0.57%) ^j^	2.16 (± 0.87%)	0.18	0.37	0.12	5.49	0.38	1.17	-	1.00
TP-7	23.29 (± 0.60%) ^ef^	3.53 (± 1.71%)	0.21	0.25	0.19	2.33	0.24	1.07	0.05	1.00
TP-8	16.73 (± 0.61%) ^i^	2.88 (± 1.96%)	0.17	0.23	0.20	2.42	0.22	1.17	0.05	1.00
TP-9	73.34 (± 0.71%) ^a^	2.13 (± 1.17%)	0.21	0.29	0.22	2.33	0.29	1.60	0.05	1.00
TP-10	49.13 (± 0.92%) ^b^	2.53 (± 1.61%)	0.20	0.28	0.22	2.20	0.31	1.38	0.07	1.00
TP-11	43.06 (± 0.54%) ^c^	2.06 (± 0.95%)	0.35	0.46	0.30	5.04	0.46	1.32	-	1.00
TP-12	22.47 (± 0.96%) ^fg^	3.08 (± 1.71%)	0.08	0.18	0.14	0.97	0.09	0.86	0.02	1.00

TPs 1–12 represent tea polysaccharides as shown in Table 1. Values represent mean ± standard deviation, and statistical analysis was carried out by ANOVA plus *post hoc* Ducan’s test, and statistical significance (*p* < 0.05) was indicated with different lowercase letters (a–k).

## Data Availability

Data is contained within the article.
